# P-381. Veterans on Cabotegravir-Rilpivirine Long Acting Treatment for HIV are Younger and More Urban Than Those Who Continue Oral ART

**DOI:** 10.1093/ofid/ofaf695.599

**Published:** 2026-01-11

**Authors:** Puja Van Epps, Brigid Wilson, Elizabeth Zink, Michael Ohl, Marissa Maier

**Affiliations:** Veterans Health Administration, Case Western Reserve University School of Medicine, Cleveland, OH; VA Northeast Ohio Healthcare System, Cleveland, Ohio; Veterans Health Administration, Cleveland, Ohio; Center for Access and Delivery Research and Evaluation CADRE, Department of Medicine, University of Iowa Carver College of Medicine, Iowa City, Iowa; VA Portland Health Care System/Oregon Health and Sciences University, Portland, Oregon

## Abstract

**Background:**

In 2021, the US Food and Drug Administration approved the first complete long-acting (LA) injectable antiretroviral therapy (ART) regimen, cabotegravir plus rilpivirine (CAB+RPV LA), for use in people with HIV (PWH) to maintain viral suppression. The Veterans Health Administration (VHA) is the largest provider of HIV care in the US and creates an opportunity to examine CAB+RPV LA uptake in a national cohort with uniform ART access. We aimed to describe demographic, temporal and geographic trends of CAB+RPV LA use in VHA nationwide.

**Figure:**
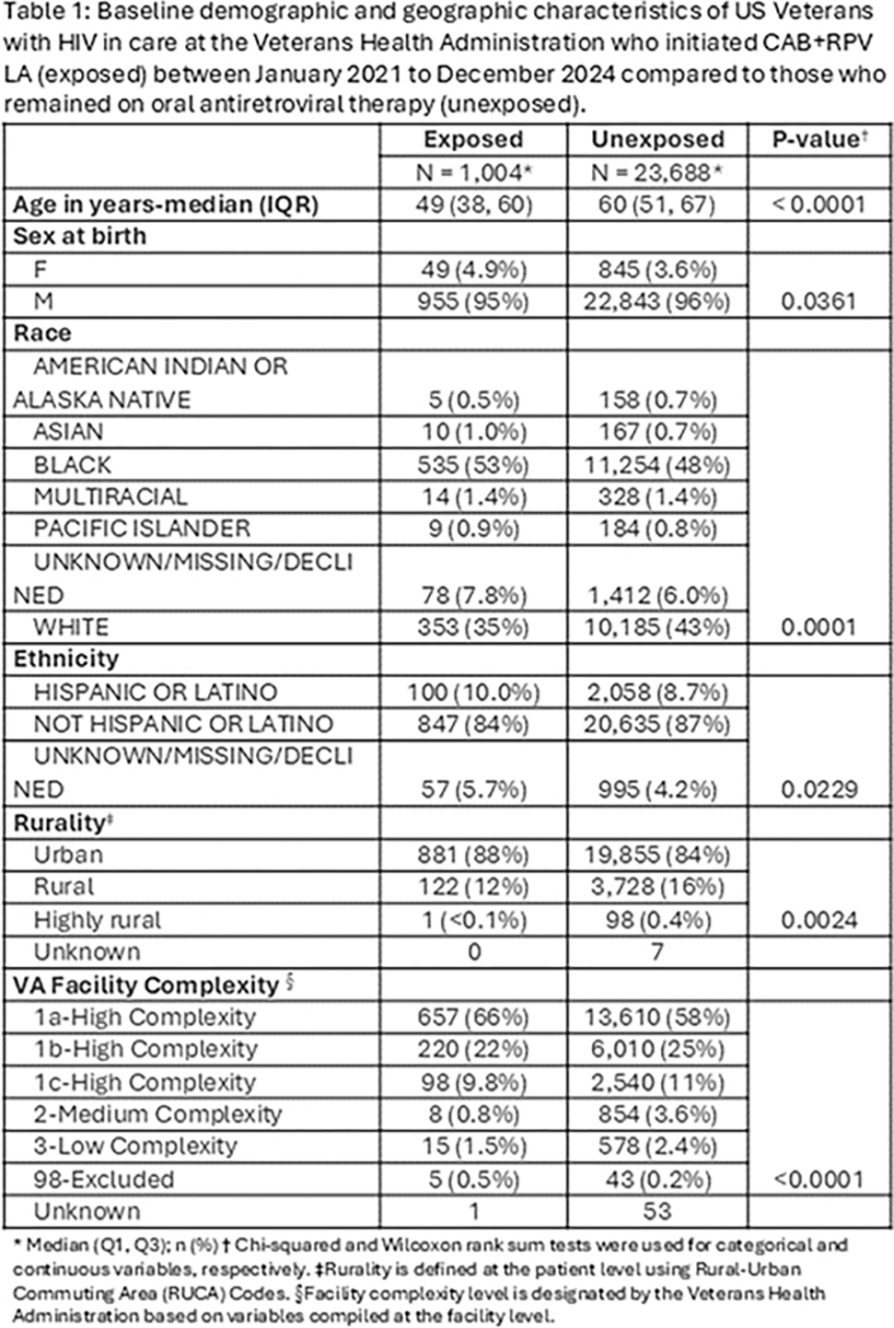

**Figure:**
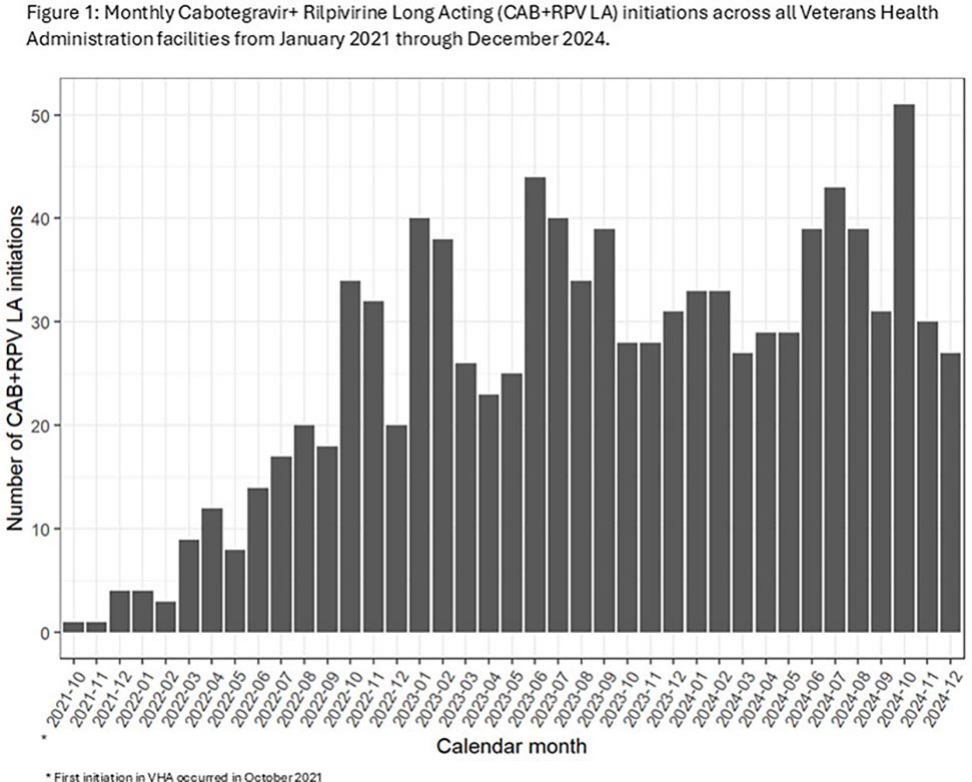

**Methods:**

Using the VHA’s Corporate Data Warehouse, we identified PWH who initiated CAB+RPV LA between January 2021 and December 2024. Those with documented receipt of at least 3 CAB+RPV LA injections were categorized as ‘exposed’. We defined ‘unexposed’ as those who did not receive CAB+RPV LA in the pre-defined study period, were virally suppressed at entry into the study and had at least one oral ART fill. We described the nationwide uptake in CAB+RPV LA, summarizing patient characteristics, and geographic trends over time. We compared the exposed and unexposed groups using Chi-squared and Wilcoxon rank sum tests and summarized the prescribing trend across states and VHA facilities.

**Figure:**
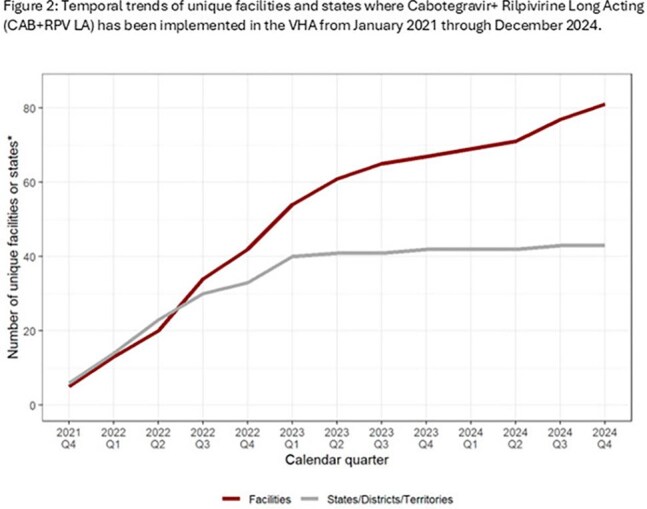

**Figure:**
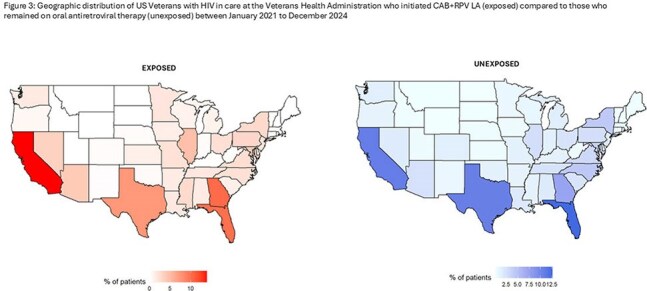

**Results:**

We identified 1004 exposed patients who initiated CAB+RPV LA from January 2021 to December 2024, while 23,688 patients were unexposed. Exposed patients were younger (mean age 49 years vs. 60 years; p< 0.001) and more likely to live in an urban area (88% vs. 84%, p=0.0024) than unexposed. The exposed group included higher proportions of Black (53% vs. 48%), Hispanic (10% vs. 8.7%) and female patients (4.9% vs. 3.6%) than the unexposed cohort. Uptake increased from 6 initiations nationally in 2021 to 441 in 2024. The number of VHA facilities that had prescribed CAB+RPV LA increased from 5 in 2021 to 81 in 2024, representing 58% of all VHA facilities treating eligible PWH. Patients at high complexity facilities, which are generally located in large metropolitan areas, were overrepresented in the exposed cohort (66% vs. 58%).

**Conclusion:**

Uptake of CAB+RPV LA has increased in the VHA since FDA approval in 2021 with greater uptake in large, high complexity, urban facilities. Further studies should evaluate barriers to implementation at smaller, rural serving clinics and older patients.

**Disclosures:**

Puja Van Epps, MD, ViiV Healthcare: Grant/Research Support

